# Development of CRISPR Cas9, spin-off technologies and their application in model construction and potential therapeutic methods of Parkinson’s disease

**DOI:** 10.3389/fnins.2023.1223747

**Published:** 2023-07-06

**Authors:** Jiangbo Qu, Na Liu, Lu Gao, Jia Hu, Miao Sun, Dongyi Yu

**Affiliations:** ^1^Center for Medical Genetics and Prenatal Diagnosis, Key Laboratory of Birth Defect Prevention and Genetic Medicine of Shandong Health Commission, Key Laboratory of Birth Regulation and Control Technology of National Health Commission of China, Shandong Provincial Maternal and Child Health Care Hospital Affiliated to Qingdao University, Jinan, Shandong, China; ^2^School of Life Science and Technology, Weifang Medical University, Weifang, Shandong, China; ^3^Institute for Fetology, The First Affiliated Hospital of Soochow University, Suzhou, Jiangsu, China

**Keywords:** Parkinson’s disease, CRISPR Cas9, model animals, precision treatment, gene editing

## Abstract

Parkinson’s disease (PD) is one of the most common degenerative diseases. It is most typically characterized by neuronal death following the accumulation of Lewis inclusions in dopaminergic neurons in the substantia nigra region, with clinical symptoms such as motor retardation, autonomic dysfunction, and dystonia spasms. The exact molecular mechanism of its pathogenesis has not been revealed up to now. And there is a lack of effective treatments for PD, which places a burden on patients, families, and society. CRISPR Cas9 is a powerful technology to modify target genomic sequence with rapid development. More and more scientists utilized this technique to perform research associated neurodegenerative disease including PD. However, the complexity involved makes it urgent to organize and summarize the existing findings to facilitate a clearer understanding. In this review, we described the development of CRISPR Cas9 technology and the latest spin-off gene editing systems. Then we focused on the application of CRISPR Cas9 technology in PD research, summarizing the construction of the novel PD-related medical models including cellular models, small animal models, large mammal models. We also discussed new directions and target molecules related to the use of CRISPR Cas9 for PD treatment from the above models. Finally, we proposed the view about the directions for the development and optimization of the CRISPR Cas9 technology system, and its application to PD and gene therapy in the future. All these results provided a valuable reference and enhanced in understanding for studying PD.

## 1. Introduction

Parkinson’s disease (PD) was reported by doctor James Parkinson before 20 decades ago, the symptoms of patients include resting tremor, gait retardation, sleep problems (presence of Paralysis agitans) (Gomperts, 2016). Later, more detailed pathological features were gradually discovered clinically, manifesting as degenerative death of nigrostriatal dopaminergic neurons, a significant decrease in dopamine neuron, and accumulation of Lewy body inclusions in the substantia nigra pars compacta (Damier et al., 1999). These lesions result in the inability of dopamine neurons in the substantia nigra region to transmit dopamine to the striatum via the substantia nigra-striatal pathway. As the second largest neurodegenerative disease in the world, PD has become the fastest growing neurological disease in the world. Its incidence increases with age, with a prevalence of more than 1% in people over 60 years old and 2–3% in those over 65 years old. It is estimated that by 2040, this disease will be expected to affect more than 12 million people (Damier et al., 1999; Dorsey et al., 2018; Dorsey and Bloem, 2018). PD not only brings physical and mental pain to the patient but also imposes a heavy burden to their families and society. Although the pathological diagnosis of PD is relatively clear at the current stage, the pathogenic mechanism of PD is not definite, and exploring the causes of PD has been a focus of neuroscience research for several decades. It is reported that both environmental factors and genetic mutations contribute to the degenerative death process of dopaminergic neurons. Currently, mitochondrial dysfunction, oxidative stress, altered protein processing, and inflammatory changes are considered to be the causes of neuronal dysfunction and death through apoptosis or autophagy (Sankhla, 2017). Aging is the most noticeable risk factor for PD, and the biochemical changes caused by aging amplify these abnormalities in the PD brain (Schapira and Jenner, 2011).

PD can be divided into sporadic and familial types, with the latter accounting for 10–15% (De Plano et al., 2022). Familial PD is usually caused by mutations in PD-related genes, including *SNCA* (Puschmann, 2013), *Parkin* (Gao and Hong, 2011; Puschmann, 2013), *PINK1* (Crosiers et al., 2011), *DJ-1* (Blauwendraat et al., 2020), *LRRK2* (van der Vegt et al., 2009), *ATP13A2* (Crosiers et al., 2011), and so on. Some PD-related genes have been identified via multiple clinical cases. Nevertheless, the pathogenesis of PD is not clarified up to now. Recently, the rapid development of CRISPR Cas9 and related gene editing technology has enabled humans to explore the relationship between genes and diseases more precisely, with more and more worldwide applications in neurodegenerative diseases such as PD. In this review, a detailed introduction to the detail and development of CRISPR Cas9, the construction of PD-related animal models, and the therapeutic methods for PD via CRISPR Cas9 and related technologies will be provided.

## 2. CRISPR Cas9 technology

### 2.1. Discovery and working principle of CRISPR Cas9 gene editing technology

**Figure 1:**
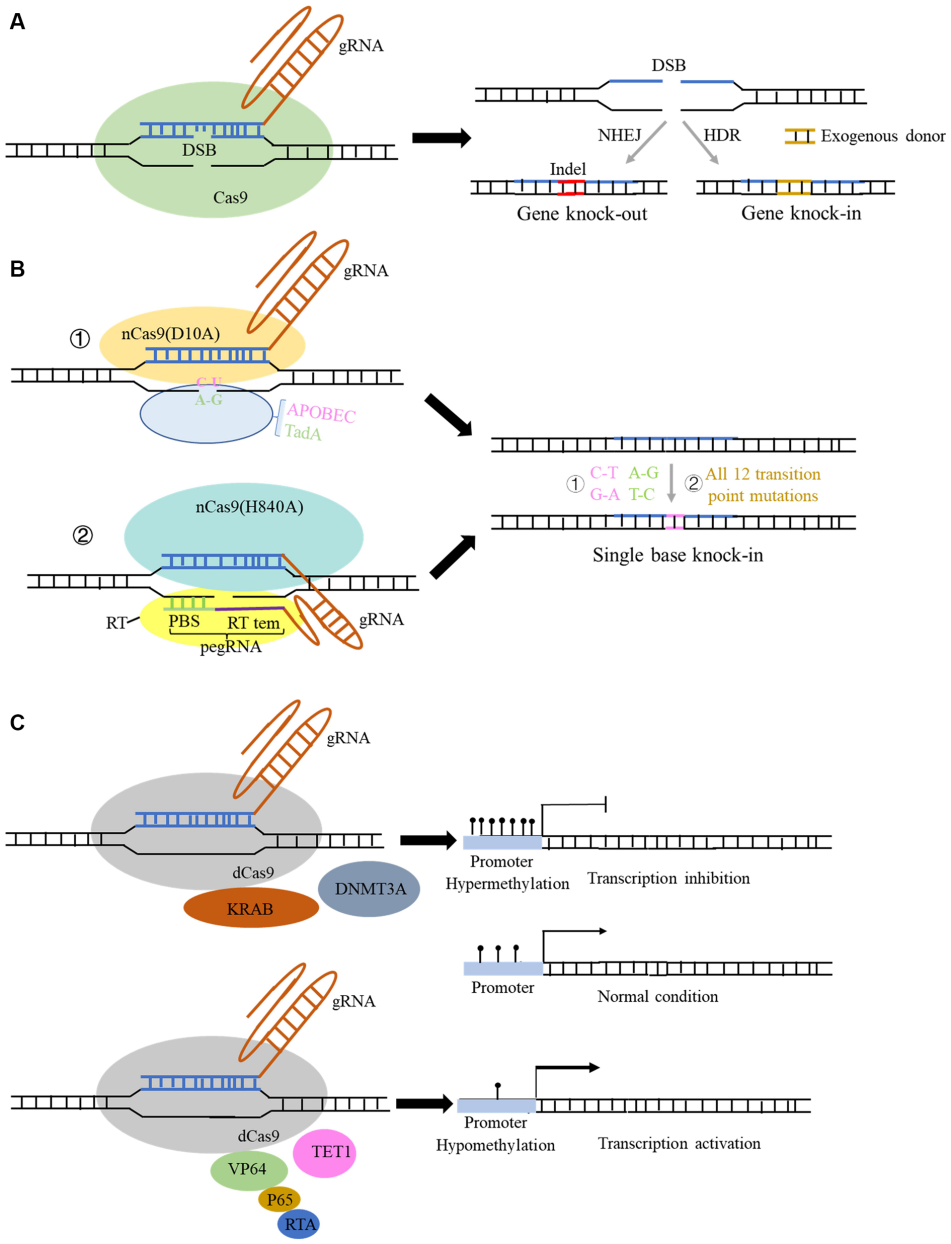
Structure and function of the CRISPR Cas9 and spin-off technology system. **(A)** Structure of the typical CRISPR Cas9 system. Genome editing using this system can be used for gene knock-out and gene knock-in with donor sequence. **(B)** Structure and function of derived CRISPR Cas9 system for single-base editing: ① cytidine base editor (CBE) could change base C to U, and adenine base editor (ABE) could change base A to G; ② prime editor (PE) could achieve all 12 transition point changes. **(C)** Structure of the derived CRISPR Cas9 technology for gene expression modulation: fusion of some transcriptional repressors or methylation transferase with dCas9 protein can repress the transcription of genes, and conversely, fusion of some transcriptional activators and demethylases with dCas9 protein can activate the gene expression.

Clustered Regularly Interspaced Short Palindromic Repeats (CRISPR) sequences were firstly found in bacteria (Ishino et al., 1987) and definitely designation in 2002 (Jansen et al., 2002). However, scientists confirmed that CRISPR sequence functions to benefit bacteria to resist viral infections in 2007 (Barrangou and Horvath, 2017). The CRISPR family contains two main types of systems, which includes several Cas proteins. The first type of system is usually found in bacteria and archaea, such as I, III, and IV Cas proteins, which function by forming multi-subunit protein-crRNA (CRISPR RNA) effector complex. The second type of system contains II, V, and VI types, which could perform target editing relying on a single crRNA-guided protein, i.e., a single multidomain protein that exercises function (Makarova et al., 2011). Herein, the second CRISPR system is more convenient to carry out the gene editing. The detail reported Cas proteins in the second system is listed in Table 1. CRISPR Cas9 is the earliest, most widespread, and the most mature technology among second CRISPR system (Makarova et al., 2017). The CRISPR Cas9 technology includes a small guide RNA (sgRNA) used to target the desired DNA molecule and Cas9 protein, a non-specific CRISPR nuclease which could cleave double-stranded DNA molecules. The Cas9 protein has two cleavage-active domains: the HNH which cleaves the DNA strand complementary to the crRNA, and RuvC domains which cleaves the non-complementary strand (Lu et al., 2021). sgRNA is composed of a trans-activating crRNA (tracrRNA) sequence that can bind to the Cas9 protein and a crRNA containing a specific sequence with about 20 nt in length that is complementary to the target sequence, and the remaining sequence of crRNA complementary to tracrRNA. Therefore, this technology searches for the motif sequence complementary to the crRNA of gRNA in the target DNA molecule with a PAM sequence behind it. Subsequently, Cas9 protein cleaves this motif and results in the formation of double-strand breaks (DSB). Afterwards, knock-out and knock-in occur via non-homologous end joining and homologous recombination in the presence of the exogenous donor sequence in cell (Figure 1A), respectively (Kaulich et al., 2015).

**Table 1:** Features of CRISPR-associated proteins.

CRISPR-associated protein name	gRNA length (nt)	Protein size (aa)	Targeted sequence length (nt)	Editing object	Effector protein domain	PAM sequence	After digestion	References
Cas9	~100	1300–1400	~20	dsDNA	HNH, RuvC	5’NGG	Blunt end	Mali et al. (2013)
Cas12a	60–70	1200-1300	19–24	dsDNA	RuvC-like	5’TTTN	Cohesive end	Paul and Montoya (2020)
Cas12b	100–120	1000-1300	15–20	dsDNA, ssDNA	RuvC-like	5’TTN	Cohesive end	Yang et al. (2016)
Cas12c1	~110	~1300	~23	dsRNA	RuvC-like	5’TTN	Cohesive end	Zhang et al. (2022)
Cas12c2	~93	~1200	~17	precrRNA	RuvC-like	5’TG	/	Huang et al. (2022) and Kurihara et al. (2022)
Cas12f	~200	400–700	~20	dsDNA, ssDNA	RuvC-like, Zn finger	5’TTTR	Cohesive end	Wu et al. (2021)
Cas12j	~60	700–800	~18	dsDNA	RuvC-like	5’TBN	Cohesive end	Pausch et al. (2020)
Cas13a/c2c2	60–70	~1250	19–24	ssRNA	2x HEPN	3’A,U,C	/	Liu et al. (2017)
Cas13b	100–120	~1150	15–20	ssRNA	2x HEPN	5′D PFS 3′NAN/NNA	/	Shmakov et al. (2017)
Cas13c	~60	~1120	22–28	ssRNA	2x HEPN	/	/	Yan et al. (2018)
Cas13d	~60	~930	22–28	ssRNA	2x HEPN	/	/	Yan et al. (2018)
Cas13X.1	~60	~775	22–28	ssRNA	2x HEPN	/	/	Xu et al. (2021)
Cas13Y.1	~60	~790	22–28	ssRNA	2x HEPN	/	/	Xu et al. (2021)

### 2.2. The evolution of CRISPR Cas9 technology

CRISPR Cas9 and related CRISPR Cas systems have been continuously optimized since their emergence, with three main areas: (1) achieving more precise single base editing, (2) reducing off-target effects, and (3) improving editing efficiency. As for the optimization of its delivery approach, the main focus is to find smaller and more portable Cas proteins, which can be effectively packaged using related lentiviral or AAV vectors and more conducive to entering into cells. To reduce off-target efficiency, scientists found that converting one of the two key amino residues in RuvC I of RuvC to alanine (D10A or H840A) could produce Cas9 nickase (Cas9n). This nickase can only cleave the DNA chain that is complementary to the crRNA and cannot cleave the non-complementary DNA chain, thus reducing off-target effects and maintaining high-efficiency gene editing. Furthermore, if the RuvC catalytic domain is mutated by point mutation (D10A) and the HNH catalytic domain is mutated by point mutation (H840A), the Cas9 protein will completely lose its nuclease activity and form dead Cas9 (dCas9), which can only bind to the target gene under the guidance of sgRNA (Qi et al., 2013).

Based on the above findings, Liu et al. fused the cytidine deaminase with nCas9 or dCas9 protein, successfully converting cytidine C to uridine U, and achieved C-to-A and corresponding G-to-T conversion in DNA replication, which was termed as cytidine base editor (CBE). Later, CBE was continuously optimized to enable efficient and permanent C:G to T:A base pair conversion in bacteria, yeast, plants, zebrafish, mammalian cells, mice, and even human embryos (Komor et al., 2016, 2017; Kim et al., 2017). Likewise, by fusing the modified adenine deaminase with nCas9 protein, the system is capable of converting adenine (A) to inosine (I) on ssDNA, and inosine (I) is recognized and read as guanine (G) during DNA replication, ultimately achieving direct substitution of A: T to G:C base pairs. This system is termed as adenine base editor (ABE) (Gaudelli et al., 2017; Komor et al., 2018; Figure 1B). The application of combination of CBE and ABE can effectively perform transition of four types of bases, whereas it is still unable to achieve transversion of the other eight types of bases as well as bases insertion and deletion. Liu team then developed the prime editor (PE) which can achieve change of 12 types situation and precise insertion (up to 44 bp) and deletion (up to 80 bp) of bases without relying on DSB and donor sequence (Anzalone et al., 2019; Figure 1B). In this system, a pegRNA was added to the 3′ end of the sgRNA, which can complement the broken target DNA 3′ end to initiate the reverse transcription. It also contains target point mutations or insertion–deletion mutations to achieve precise base editing (Figure 1B). Later, many studies innovated and optimized single-base editing, and three teams successfully achieved simultaneous CBE and ABE in a single gene editing, which develop a new double-base gene editor (Grünewald et al., 2020; Sakata et al., 2020; Zhang et al., 2020). Yang et al. recently developed a new adenine base editor (AYBE), which can firstly achieve the transversion of adenine (Tong et al., 2023b).

The spin-off technologies of the CRISPR system are not only focused on the optimization of editing DNA bases, but in the regulation of gene expression without changing the genome sequence. DNA editing directly modifies genome sequence permanently and has potential off-target probability, which poses serious clinical application risks. While the regulation of gene expression is usually mild and reversible, and its application in disease treatment can also make up for the shortcomings of DNA editing. The existing CRISPR systems for regulating gene expression include RNA editing systems of Cas13-related proteins (Table 1) and DNA modification regulation systems in which dCas9 protein is coupled with various regulatory factors (Figure 1C). Binding dCas9 to the transcription start site (TSS) of a gene can block the start of transcription, thereby inhibiting gene expression (Figure 1C). Binding dCas9 coupled with transcriptional suppressors or activators can inhibit or activate downstream target gene transcription, respectively (Sen and Thummer, 2022). Fusing some methyltransferases and demethylases to dCas9 protein can selectively regulate the DNA methylation of gene promoter, regulating gene expression (Figure 1C). Cas 13 family is an RNA-dependent RNA endonuclease (Table 1). It can specifically cleave the target RNA to inhibit gene expression in the presence of a PFS sequence in target RNA. Cas13 proteins mainly includes four subtypes, namely CRISPR-Cas13a, b, c, d (Abudayyeh et al., 2017). Among them, CasRx (RfxCas13d) has received widespread attention for its higher efficiency, lower off-target rate. More importantly, Cas13d has a smaller size compared to other family members, which can be more easily packaged into viral vectors, thus having better delivery advantages and application prospects (Kushawah et al., 2020). Cas13d has been reported to achieve effective gene silencing in mouse liver (He et al., 2020). However, research has also found that the CRISPR-Cas13 editing system has significant collateral degradation effects while cleaving RNA sequences, that is, after Cas13 binds to the target RNA and is activated, it can degrade bystander RNAs to some extent. To address this problem, Yang et al. designed a dual fluorescence reporting system to detect collateral effects in mammalian cells and seek for Cas13 variants (Tong et al., 2023a). The team found that the mutated Cas13 variants Cas13d-N2V8 and Cas13X-M17YY had virtually eliminated collateral effects when editing RNA. In addition, researchers have also discovered two new proteins in the Cas13 protein family, termed as Cas13x and Cas13y, and developed a new RNA editing technology based on them. CRISPR Cas13x and CRISPR Cas13y have stronger knockdown activity than previous Cas13a and Cas13b system, and some derived truncated types can achieve RNA single-base editing after fusion with RNA single-base enzymes (Xu et al., 2021; Table 1). Yang et al. also developed a new CRISPR-Cas12f system (enOsCas12f1 and enRhCas12f1) in mammalian cells with high activity, broad targeting range, and high fidelity. After removing the cleavage activity, denOsCas12f1 is an epigenetic editor and gene expression activator (Kong et al., 2023), which shows strong regulatory activity in mammalian cells.

## 3. Pathogenic insights from PD-related models

Although the pathogenesis of PD is not yet understanding, the phenotype is clear. It is not possible to study the pathogenesis of PD and observe the disease process in humans due to the limitations of medical ethics and experimental risks. It is urgent to simulate the pathogenic biological PD model from the perspective of the pathogenesis and development process of the disease. In the next part, we will introduce the pathogenic insights from PD-related models including cells, small model animals, and large mammal animals (Table 2).

**Table 2:** Overview of the studies on PD models using CRISPR Cas9 and related editing strategies.

PD model	Target gene	Target variation location	Delivery methods	Main results	References
iPSC and hESC	LRRK2	p.G2019S	Transfection of helper-dependent adenoviral vectors	Pathological changes in the nucleus	Liu et al. (2012)
iPSC from PD patient	LRRK2	p.Gly2019Ser	/	Delayed mitochondrial autophagy and impaired cellular respiration and metabolism	Hsieh et al. (2016)
iPSC from PD patient	PARK2	Homozygous deletion of parkin exons 2–4	/	Increased oxidative stress accompanied by activation of the Nrf2 pathway; Abnormal mitochondrial morphology and impaired mitochondrial turnover	Imaizumi et al. (2012)
iPSC from PD patient	PARK2	Homozygous deletion of exons 6 and 7	/		
Human dopaminergic SH-SY5Y	UQCRC1	p.Ala25Glyfs*27	Electrotransfection of CRISPR Cas9 plasmid	Axonal degeneration and mitochondrial respiratory chain dysfunction in cells	Lin et al. (2020)
Drosophila	UQCRC1	p.Tyr314Ser	Injection of CRISPR Cas9 system to Oregon R embryos	Age-dependent locomotor defects, dopaminergic neuronal loss, peripheral neuropathy, impaired respiratory chain complex III activity and aberrant mitochondrial ultrastructures in nigral neurons	Lin et al. (2020)
Mouse	UQCRC1	p.Tyr314Ser	Injection of CRISPR Cas9 system to one-cell stage embryos		
Drosophila	tango14	p. Gly217Ter	Injection of CRISPR Cas9 system to pre-blastoderm embryos	Shortened life span, cholesterol accumulation in dopaminergic neurons	Xue et al. (2022)
Zebrafish	DJ-1	Frame shift from exon1	Injection of CRISPR Cas9 system to one-cell stage embryos	Lower tyrosine hydroxylase levels, skeletal muscle respiratory failure, and lower body weight	Edson et al. (2019)
Zebrafish	GCH1	p.T59Rfs27*	Injection of CRISPR Cas9 system to one-cell stage embryos	Monoamine neurotransmitter deficiency, motor deficit at 8dpf, death at 12dpf	Larbalestier et al. (2022)
Zebrafish	ATP12A2	Frame shift from exon2	Injection of CRISPR Cas9 system to one-cell stage embryos	Significant reduction in the number of TH+ neurons in the posterior nodes and locus ceruleus	Nyuzuki et al. (2020)
Mouse	VPS35	p.D620N	Injection of CRISPR Cas9 system to pronuclei of one-cell embryos	Survival disadvantage, and DA release is significantly reduced in caudate-putamen	Ishizu et al. (2016)
Mouse	CDK5	/	Stereotactic injection of the CRISPR Cas9 system into the dorsal striatum	Deficits in locomotor activity and disturbances in activity/rest behavior, and downregulation of dendritic length and decreased number of functional synapse in the brain	Zhou et al. (2022)
Mouse	PNPLA9	p.R748W	Injection of CRISPR Cas9 system to one-cell stage embryos	Exercise disorders and accumulation of peroxisomal phospholipids	Sun et al. (2021)
Mouse	prkn/pink1/dj-1	/	Editing ES cells by homing endonuclease technology combined with transfection and then transplanting ES into blastocysts	No obvious neuronal loss, normal behavior	Kitada et al. (2009)
Pig	SNCA	p.E46K, p.H50Q, p.G51D	Transfection of CRISPR Cas9 system into fbroblasts through Xfect and then performing SCNT	No obvious neuronal loss, normal behavior	Zhu et al. (2018)
Pig	PARK7	/	Transfection of TALEN system into primary fetal fibroblast cells through electroporation and then performing SCNT	DJ-1 protein was repressed in all the detected tissues and all pigs die due to due to cloning defect	Yao et al. (2014)
Pig	PARK2 and PINK1	First exon	Transfection of CRISPR Cas9 system into fbroblasts through electroporation and then performing SCNT	No obvious neuronal loss, normal behavior	Zhou et al. (2015)
Pig	Parkin/DJ-1/PINK1	/	Injection of CRISPR Cas9 system to pronuclei of one-cell embryos	No obvious neuronal loss, normal behavior	Wang et al. (2016)
Monkey	PINK1	/	/	Some died after birth, and surviving monkeys showed severe degeneration and death of neural cells in the brain	Yang et al. (2019b)
Monkey	PINK1	Frame shift mutation	Injection of CRISPR Cas9 system to pronuclei of one-cell embryos	No obvious neuronal loss,normal behavior	Chen et al. (2021)
Monkey	PINK1 and DJ-1	Second and third exons of PINK1, second and fourth second exons of DJ-1	Stereotactic injection of AAV9-Packaged CRISPR Cas9 into specific brain regions	Severe loss of dopaminergic neurons and accumulation of pathology of a-synaptic nucleoprotein in the substantia nigra	Li et al. (2021)

### 3.1. Construction of PD models from cell-level

Some studies have constructed models by induced pluripotent stem cells (iPSCs) from somatic cells of PD patients with clear genetic mutations that cause the disease. The iPSCs were differentiated into neural stem cells which can simulate the relevant phenotypes of neuronal cells in the brain of PD patients. Mutations in some genes have been found in both familial and sporadic PD cases, such as the *LRRK2*, *SNCA*, and *PARKIN* genes (Satake et al., 2009; Simón-Sánchez et al., 2009; Nalls et al., 2011). Therefore, using cell models with these genetic mutations is more likely to reveal the pathogenesis of PD. Researchers found that neural stem cells differentiated from iPSC cells of patient with *LRRK2* p.G2019S mutation showed increased susceptibility to proteasome stress, and had transgenerational defects in nuclear envelope organization, clonal expansion, and neuronal differentiation (Liu et al., 2012). Subsequently, using knock-in technology to change the point mutation to wild-type, the above phenotype can be reversed, which proposed that nuclear morphology alteration as a clinical diagnostic feature of PD. Further research found that LRRK2 forms a complex with a mitochondrial outer membrane protein Miro in iPSC cells, which then removes Miro. Once the removal of Miro is affected in LRRK2 p.G2019S mutation cells, it will delay mitochondrial autophagy, impairing cell respiration and metabolism (Hsieh et al., 2016). Mitochondrial dysfunction is a key aspect of Parkinson’s disease, and mutations in the *PRKN*/*PARK2* gene have been reported to be associated with early-onset familial PD. This gene can regulate mitochondrial function and autophagy processes (Narendra et al., 2008). Researchers edited iPSCs cells from two PD patients with *PARK2* gene mutations and found that neurons differentiated from iPSCs showed increased oxidative stress and enhanced Nrf2 pathway activity. In addition, neural cells showed abnormal mitochondrial morphology and impaired mitochondrial homeostasis (Imaizumi et al., 2012). In another study, scientists identified multiple pathogenic mutations in *UQCRC1* in both familial and sporadic PD patients. After knock-in of these mutations in human dopaminergic SH-SY5Y cell lines, axonal degeneration and mitochondrial respiratory chain dysfunction were found (Lin et al., 2020).

### 3.2. Construction of PD models with small model animals

*PINK1* and *Parkin* form a central signaling axis that plays an important role in controlling the mitochondrial autophagy process in dopaminergic neurons. In *pink1* null Drosophila, knocking down *UCHL1* gene using RNAi can rescue the PD-related pathogenesis. Specifically, the loss of UCH deubiquitination promotes mitochondrial autophagy by activating the expression of *AMPK* and *ULK1* (Ham et al., 2021). In Drosophila, researchers used CRISPR-Cas9 to knock out *tango14*, the homologous gene of *NUS1*, and found that the lifespan of Drosophila was shortened, and cholesterol accumulation appeared in dopaminergic neurons, which confirms that this gene is associated with the occurrence of PD due to lipid metabolism abnormalities (Xue et al., 2022).

In zebrafish, researchers constructed a *dj-1* null strain targeting exon 1 using CRISPR Cas9. It was found that *dj-1*^−/−^ zebrafish developed normally in the early stages, but showed lower levels of tyrosine hydroxylase, skeletal muscle respiratory failure, and lower body weight as development progressed (Edson et al., 2019). Proteomic analysis of the brains from *dj-1*^−/−^ zebrafish revealed downregulation of proteins related to mitochondrial metabolism, autophagy, stress response, redox regulation, and inflammatory response. Then, researchers developed a new, unbiased computational method to classify the movement disorders of adult *dj-1*^−/−^ zebrafish (Hughes et al., 2020). Mitochondrial calcium uniporter (*MCU*) participates in mitochondrial dysfunction and cell death caused by excitotoxicity, inflammation, and oxidative stress by regulating mitochondrial calcium uptake, and plays an important role in PD (Liao et al., 2017). Soman et al. generated a zebrafish model with double knockout of *mcu* and *pink1* using the CRISPR-Cas9 system. Compared with *pink1*^−/−^ zebrafish, (*pink1*; *mcu*)^−/−^ zebrafish showed a higher number of dopaminergic neurons and a higher mitochondrial membrane potential. In addition, mitochondrial sphericity was restored, and animals were protected from PD MPTP neurotoxin. Therefore, *mcu* may be an effective target for the treatment of PD (Soman et al., 2019).

Mutations in GTP cyclohydrolase 1 (*GCH1*) may lead to the development of PD. In zebrafish, after knocking out *gch1* using CRISPR Cas9 technology, zebrafish showed monoamine neurotransmitter defects, movement defects at 8 days post fertilization (dpf), and death at 12 dpf. Tyrosine hydroxylase (Th) protein was up-regulated, but there was no loss of dopaminergic (DA) neurons (Larbalestier et al., 2022). *ATP12A2* is an autosomal recessive pathogenic gene for juvenile PD, also known as Kufor-Rakeb syndrome. *Atp13a2*^−/−^ zebrafish were also established by CRISPR Cas9 gene editing. The number of TH^+^ neurons in the posterior lobe and the locus coeruleus of *atp13a2*^−/−^zebrafish was significantly reduced, indicating dopaminergic neuron degeneration, lysosomal dysfunction, and intracellular transport disorders (Nyuzuki et al., 2020). *PARL*, which encodes presenilin-associated rhomboid-like protein (PARL), has been found to contribute to mitochondrial morphology, function and is associated with familial PD. PARL is mitochondrial inner membrane protease that acts on many mitochondrial proteins involved in mitochondrial morphology, apoptosis, and mitophagy. After knocking out this gene in zebrafish using CRISPR Cas9, dopamine neurons were lost in the brain, and tyrosine hydroxylase transcription levels decreased, leading to impaired olfaction and decreased motor parameters in zebrafish (Merhi et al., 2021).

Mouse has been widely used to construct models related to PD. Researchers have generated a model called MCI-Park model in mice by CRISPR Cas9 to produce dopamine neurons that lack *NDUSF2*, which encodes mitochondrial complex I. Mice lacking *NDUSF2* showed neurodegenerative changes (González-Rodríguez et al., 2021). Point mutations in the vacuolar protein sorting 35 gene (*VPS35*) are associated with an autosomal dominant late-onset PD (PARK17). Homozygous deletion of *Vps35* by CRISPR Cas9 resulted in survival disadvantage and significantly reduced DA release in the caudate putamen of adult homozygous *Vps35* mutant mice (Ishizu et al., 2016). Cyclin-dependent kinase 5 (*CDK5*) negatively regulates dopamine signaling in the striatum, playing a critical role in circadian rhythm disruption and sleep disorders. After knocking out *Cdk5* using CRISPR Cas9, mice showed defects in motor activity and interference with activity/rest behavior, and the number of dendritic length and functional synapses in the mouse brain were down-regulated (Zhou et al., 2022). Peroxidized phospholipids caused by ferroptosis have been reported to be associated with the onset of some PD cases. PNPLA9, as a hydrolytic enzyme, can preferentially hydrolyze peroxidized phospholipids. In mice, knocking out *Pnpla9* resulted in progressive PD motor disorders and accumulation of peroxidized phospholipids (Sun et al., 2021). Although gene editing of several genes can simulate some symptoms of PD, editing classic genes related to PD has not been successful in mice. Even if simultaneously knocking out three hot genes related to PD, including *Prkn*/*Pink1*/*Dj-1*, no neurodegenerative phenotype was observed in mice, even in older mice (Kitada et al., 2009). This suggests that these hot genes related to PD may have functional differences in the nervous systems between humans and mice.

### 3.3. Construction of PD models with large mammal animals

In spite of the fact that gene editing in small rodents is convenient, it is difficult to reproduce significant neurodegeneration including loss of neurons as PD patients (Deng and Siddique, 2000; Deng et al., 2018). Therefore, it is not conductive to using mice to find therapeutic methods for PD. Recently, some large animal models of PD have been successfully constructed (Yang W. et al., 2021). These animal models are able to better simulate the phenotype of human PD, providing new insights into the pathogenesis of PD. Although the emotional and cognitive abilities of pigs are not as similar to those between monkeys and humans, their brain structure is roughly similar to that of humans. In addition, pigs reach sexual maturity (around 6 months) earlier than monkeys, have a shorter gestation period (about 4 months), and give birth to over 10 offspring in one litter. Endogenous genes in pigs can be edited through CRISPR Cas9 combined with somatic cell nuclear transfer (SCNT) to produce knock-out or knock-in models. All of these advantages make pigs a promising alternative large animal model for investigating human diseases (Lunney et al., 2021). Researchers used CRISPR Cas9 and SCNT technology to establish a Bama miniature pig models with *Snca* missense mutations (p.E46K, p.H50Q, p.G51D). However, PD-specific phenotype was not present such as immunopositivity for SNCA and loss of dopamine neurons in the substantia nigra (Zhu et al., 2018). TALEN combined with SNCA was utilized to construct *Park7* knock-out pigs, these pigs were all dead after birth and DJ-1 protein was found significantly inhibited in various tissues (Yao et al., 2014). Another study used CRISPR Cas9 combined with SCNT to prepare *Park2* and *Pink1* double knock-out pigs, however, like the mouse model, the double knock-out pigs also showed no clinical signs of PD at 7 months old (Zhou et al., 2015). Wang et al. (2016) generated triple knock-out (*Parkin*, *Dj-1*, and *Pink1*) Bama miniature pigs, but the piglets did not exhibit the PD clinical phenotypes at 10 months of age. Considering the higher similarity of brain structure between monkey and human, gradual studies have been conducted using monkey as PD models in recent years. Li et al. performed the first knock-out of *pink1* in monkey and found that knocking out *pink1* gene resulted in part of monkey died and remaining monkeys displayed severe degeneration and death of neural cells in the brain (Yang et al., 2019a,b). Subsequently, researchers found *Pink1* genes was expressed at a very low level in mice, whereas at a high level in primate brain tissues, suggesting it is unique to human or primates. This may explain the reason behind the inconsistent results of gene editing of *Pink1* in mouse and monkey. Moreover, *Pink1* was found not aggregating in mitochondria, but rather in the cytoplasm. Under non-stressful conditions, *Pink1* functions to phosphorylate relevant neuronal proteins in the brain, protecting neurons from damage (Yang et al., 2022). It challenged the previous notion that *Pink1* mutation causes PD due to mitochondrial dysfunction and autophagy blockade, suggesting the importance of developing drugs targeting protein phosphorylation in treating PD due to *Pink1* mutation. Chen et al. developed a modified and optimized CRISPR Cas9n to knock out *Pink1* gene in embryonic cells of Cynomolgus monkey. But the monkeys did not show a phenotype of neurodegenerative disease (Chen et al., 2021). Nevertheless, this study did not detect the expression of *Pink1* in the brain, so it is not clear whether the lack of effect is due to editing heterozygosity. In another research, stereotactic injection of AAV to deliver CRISPR Cas9 system targeting *Pink1* and *Dj-1* to the monkey brain, severe loss of dopaminergic neurons in the substantia nigra and accumulation of a-synuclein pathology were observed (Li et al., 2021). Hence, once the expression of *Pink1* in the brain, especially in the substantia nigra, is knocked out in monkeys, the development of loss of dopamine neurons in the substantia nigra should be a clear consequence.

## 4. Attempts and effects of CRISPR Cas9 in treating PD

**Figure 2:**
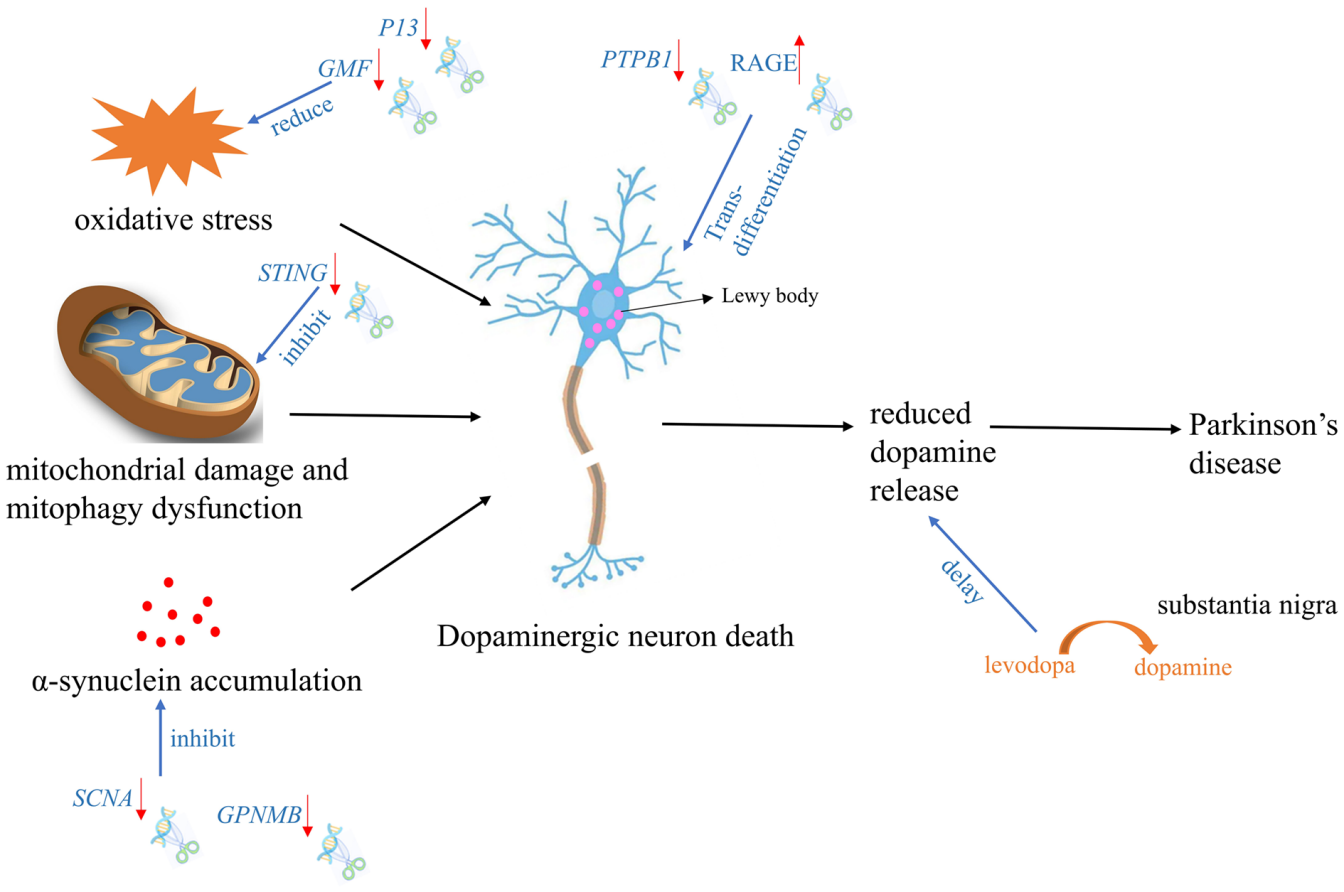
Targeted gene therapy using CRISPR Cas9 and spin-off technologies during PD development process. The red arrow indicates the expression tread of the target gene after gene editing.

According to the reported literature, the use of gene editing technology for the treatment of PD can be mainly divided into two categories: one is intervention and treatment during the formation process of PD, and the other is conversion of other types of neural cells into dopaminergic neurons (Kantor et al., 2018; Zhou et al., 2020). Intervention in the formation of PD is mainly carried out through editing of genes related to mitochondrial damage and autophagy, SNCA accumulation, and stabilization of oxidative phosphorylation (Figure 2).

### 4.1. Results *in vitro* cell lines

Scientists have designed a one-step lentiviral vector targeting *Snca* intron 1, which fused the catalytic domain of DNA methyltransferase 3A (DNMT3A) with dCas9 to carry out gene regulation (Wüllner et al., 2016; Kantor et al., 2018). Application of this system to hiPSC-derived dopaminergic neurons from PD patients with SNCA triploidy resulted in a fine down-regulation of SNCA mRNA and protein. Furthermore, reduced SNCA protein level by CRISPR-dCas9-DNMT3A system rescued disease-related cellular phenotypic features of SNCA triplicated dopaminergic neurons, such as mitochondrial ROS production and low cell viability (Wüllner et al., 2016). This suggested that combining the regulatory region sequences of genes with CRISPR-dCas9 technology may become a new epigenetic-based treatment method for PD. Apart from iPSC cells, human embryonic stem cells (hESCs) have also been used for research. Chen et al. have used CRISPR-Cas9n editing to remove *SNCA* gene from hESCs. These stem cells were then able to be converted into dopamine-producing neuronal cells *in vitro*, and were sensitive to the pS129-αSyn positive SNCA protein aggregates without forming toxic clumps. This study may benefit young patients with PD and patients with aggressive PD (Chen et al., 2019).

In BV2-G cells, knocking out of *GMF* significantly reduced oxidative stress by reducing ROS production and calcium flux. Furthermore, *GMF* deficiency significantly reduced nuclear translocation of NRF2, which regulates HO-1 and ferritin activation, the expression of cyclooxygenase 2 (*COX2*) and nitric oxide synthase 2 (*NOS2*) in BV2 microglia. GMF may regulate iron metabolism by regulating NRF2-HO1 and ferritin expression, thereby mediating protein aggregation in microglial cell homeostasis associated with progression of PD (Selvakumar et al., 2019).

### 4.2. Results in animal models

In a PD rat model with overexpression of SNCA (p. A53T), the significant rescue of α-synuclein overexpression, reactive astrogliosis, dopamine neuron degeneration, and Parkinsonian motor deficits was observed after knocking out *SNCA* gene using CRISPR Cas9 (Yoon et al., 2022). GWAS analysis of the chromosome 7p locus associated with SNCA identified an interaction between non-metastatic melanoma protein B (*GPNMB*) and *SNCA*. It furtherly showed that absence of *GPNMB* resulted in the loss of the ability to internalize α-synuclein fibrils and develop α-synuclein pathology in iPSC-derived neurons (Diaz-Ortiz et al., 2022). In addition, compared to 59 healthy individuals, GPNMB was elevated in the plasma of 731 Parkinson’s patients. And these Parkinson’s patients had more severe symptoms, indicating that *GPNMB* is a novel PD risk and biomarker, and may serve as a potential target for PD treatment.

*P13* is a novel protein involved in mitochondrial oxidative phosphorylation and its overexpression can induce mitochondrial dysfunction and cell apoptosis. Knocking out *P13* can alleviate toxin-induced mitochondrial dysfunction and apoptosis in dopaminergic SH-SY5Y cells. Furthermore, researchers constructed a mouse strain with heterozygous deletion of *P13* and found that heterozygous knock-out of *P13* prevented toxin-induced motor deficits and loss of dopamine neurons in the substantia nigra (Inoue et al., 2018). These results indicated that the regulation of P13 may be a new target for therapeutic interventions in PD. In *Prkn*^−/−^ mice, exhaustive exercise leaded to inhibition of mitochondrial autophagy and a significant increase in the levels of inflammatory factors in the serum. Notably, knocking out *STRING* gene via CRISPR Cas9, the phenotype can be rescued (Sliter et al., 2018), which suggested that *STRING* is a target gene that regulates mitochondrial stability in PD’s patients with *PRKN* mutation. After knocking out *NDUSF2* which is responsible for formation of mitochondrial complex I, mitochondria were damaged but neurons remained intact for a relatively long time in mice. Further studies revealed that these neurons could release dopamine through the cytosol and dendrites in the nigrostriatal fraction to maintain specific motor functions, despite the absence of dopamine in the striatum (González-Rodríguez et al., 2021). Researchers subsequently designed gene therapies targeting the substantia nigra, which enables neurons in the substantia nigra to convert levodopa to dopamine, these facilitates improved treatment with levodopa in the advanced stages of PD.

### 4.3. Results of cell trans-differentiation

Recently, some teams have proposed an ideal strategy for endogenous neuronal regeneration via *in situ* trans-differentiation of glial cells into neurons. Using CRISPR Cas9, cord blood-derived mesenchymal stem cells (UCB-MSC) could secret soluble RAGE. After transplantation of these UCB-MSC into striatum of PD mice, the mice showed a significant reduction in neuronal cell death and increased motility in striatum and substantia nigra (Lee et al., 2019). Qian et al. (2020) and Zhou et al. (2020) treated PD model mice by editing PTBP1 using shRNA and CRISPR CasRx system, respectively. After suppressing the expression of PTBP1 in astrocytes, these cells can efficiently trans-differentiate *in situ* into functional neurons within weeks to months, which improves the motor function of PD mouse models. While a recent repeat experiment of knocking down PTBP1 with shRNA packaged by AAV did not obtain the similar results. Neither signs of trans-differentiation of astrocytes to neurons nor increasing the number of neurons or decreasing the number of astrocytes was observed over half a month to 3 months. Furthermore, down-regulation of *PTBP1* in astrocytes did not improve cognitive function, reduce synaptic damage and Aβ/tau pathology in AD model mice (Guo et al., 2022). This suggested the demand for a more systematic, rigorous and stable strategy for future research on trans-differentiation of central nervous system (CNS) cells.

## 5. Limitations and improvement of CRISPR Cas9 technology

Although CRISPR Cas9 has been studied in some model animals and provided new insights into the mechanisms of diseases, there are still limitations and areas for improvement. Constructing non-human primate models with CRISPR Cas9 and spin-off gene editing technologies is a trend in studying the neural system PD in the brain, but the high cost of materials, long breeding cycles, and difficulty of gene editing operations make it difficult to form large-scale studies like mice. In addition, CRISPR Cas9 technology is prone to mosaic individuals, which can be eliminated in hybrid offspring but it can be time-consuming. Meanwhile, the current CRISPR Cas9 system still suffers from low gene editing efficiency and the presence of off-target effects (Zhang et al., 2015; Liu et al., 2018). Several studies have explored to improve the gene editing efficiency of CRISPR Cas9 on various parameters, such as controlling the GC content to 40–60% (Ren et al., 2019) and using multiple sgRNAs targeting the same gene (Lin et al., 2014). In addition, researchers have designed several variants of Cas9 protein to down-regulate off-target effects, such as HypaCas9, nSpCas9, snipper-Cas9, xCas9, etc. (Chen et al., 2017; Naeem et al., 2020). At the same time, the optimization of CRISPR delivery system is also an issue that must be improved to bring it to clinical application. The current delivery system of CRISPR cas9 is divided into physical delivery, chemical delivery, and viral delivery methods. Physical delivery includes electrotransfection, microinjection, and stereotaxic injection, which have the advantage of not integrating exogenous genomic material into the host cell and low immunogenicity (Han et al., 2020), but the technique is unable to edit all the histiocyte (Li et al., 2020). Calcium phosphate and liposome mediated transfection have the same advantages as physical methods. Nevertheless, chemical delivery methods are also not applicable to whole organisms and delivery efficiency is low (Han et al., 2020). Viral delivery systems, combining adenoviruses, lentiviruses, and adeno-associated viruses, are highly efficient, persistent and can infect dividing and nondividing cells (Yang Y. et al., 2021). However, their common drawbacks are limited packaging capacity and existing immunogenicity (Han et al., 2020). Furthermore, lentiviruses are not suitable for *in vivo* gene therapy due to integration of host and exogenous nucleic acid. Recently, novel nanoparticles have emerged as delivery tools for *in vivo* gene therapy (Borbolla-Jiménez et al., 2021). To date, nanoparticles in CRISPR delivery possess some advantages including easy synthesis, high efficiency, low cost, adjustable size, non-mutagenicity, and non-immunogenicity (Yan et al., 2021).

## 6. Conclusion and prospectives

The manipulation CRISPR Cas9 and derivative technologies to study PD diseases has now become a common strategy, which can be applied to construct gene editing cellular and animal models. These models can be used not only to verify the pathogenicity of PD-related disease causative genes, but also to find appropriate interventions and therapeutic approaches. In this review, we first introduced the development process of CRISPR Cas9 and derivative technologies, among which, we believe that CRISPR dCas9 and CRISPR Cas13 series may be more promising for human gene editing therapy in the future. Because the former does not cut the DNA strand, and the latter only cuts RNA strand, both are relatively safe. Subsequently, we introduced PD-related models including *in vitro* cell and various animal models constructed using CRISPR Cas9 and spin-off technologies. Among these models, large animal models especially non-human primate models of PD pathogenicity show more advantages in terms of efficiency and success in mimicking human PD manifestations and seeking for the therapeutic methods. More non-human primate models of PD will be constructed, and the improvement of CRISPR Cas9 technology, including the reduction of vector size and off-target rate, the improvement of cutting efficiency and the optimization of delivery system, are the aspects that need to be improved in the future. Our view is that in the future, it would be more meaningful to inject optimized CRISPR Cas9 systems wrapped in nanomaterials or adenoviruses into specific brain regions in non-human primates using stereotactic injection techniques. On the one hand, this method reduces the time from editing the fertilized egg to obtaining the phenotype, and on the other hand, direct injection attempts into brain regions have greater clinical relevance, considering that some clinical experiments have successfully injected dopamine or nutrient factors into specific brain regions of PD patients with good results. Therefore, improving the delivery efficiency of the CRISPR Cas9 system and reducing the negative effects on the brain in optimized stereotactic injection experiments will be important aspects to its application in gene-editing for PD treatment.

## Author contributions

JQ wrote the manuscript with support from NL, LG, JH, MS, and DY. All authors contributed to the article and approved the submitted version.

## Funding

This work was funded by projects of medical and health technology development program in Shandong province (202201030960).

## Conflict of interest

The authors declare that the research was conducted in the absence of any commercial or financial relationships that could be construed as a potential conflict of interest.

## Publisher’s note

All claims expressed in this article are solely those of the authors and do not necessarily represent those of their affiliated organizations, or those of the publisher, the editors and the reviewers. Any product that may be evaluated in this article, or claim that may be made by its manufacturer, is not guaranteed or endorsed by the publisher.
